# Study on Dynamic Recrystallization Behavior and Numerical Simulation Prediction of Martensite Stainless Steel 04Cr13Ni5Mo

**DOI:** 10.3390/ma18174047

**Published:** 2025-08-29

**Authors:** Tonghui Sun, Huiqin Chen, Ruxing Shi, Bo Zhang, Hongqiang Shi

**Affiliations:** 1 School of Materials Science and Engineering, Taiyuan University of Science and Technology, Taiyuan 030024, China; sunth2009@126.com; 2 Luoyang Zhongchong Casting and Forging Co., Ltd., Luoyang 471000, China; 15038647370@163.com (R.S.); zhangbo32103@126.com (B.Z.); 18703855514@163.com (H.S.); 3 CITIC Heavy Industries Co., Ltd., Luoyang 471000, China

**Keywords:** 04Cr13Ni5Mo, martensitic stainless steel, dynamic recrystallization, microstructure, finite element simulation

## Abstract

To address the coarse and mixed grain phenomena in ultra-large martensitic stainless steel forgings, this study investigated the hot deformation behavior of 04Cr13Ni5Mo martensitic stainless steel under deformation conditions of 950–1200 °C and strain rates of 0.001–0.1 s^−1^ using Gleeble-1500D thermomechanical simulation tests. Based on the experimental data, the flow stress curves of the steel were obtained, and a dynamic recrystallization (DRX) kinetic model was established. The model was then integrated into finite element software for simulation to verify its reliability, providing theoretical guidance for optimizing high-temperature forging processes. The results demonstrate that dynamic recrystallization in 04Cr13Ni5Mo steel occurs more readily at temperatures above 1050 °C and strain rates below 0.1 s^−1^. Under the selected hot compression test condition (1100 °C/0.01 s^−1^), the simulated grain size in the central deformation zone was 48.98 μm, closely matching the experimentally measured value of 48.18 μm. This agreement confirms the reliability of finite element-based prediction and control of grain size in martensitic stainless steel forgings.

## 1. Introduction

The alloy 04Cr13Ni5Mo is a low-carbon martensitic stainless steel with chromium (Cr), nickel (Ni), and molybdenum (Mo) as its primary alloying elements. It is also known as ASTM A182 F6NM, which has a similar composition to ASTM A743 CA6NM, widely used for hydropower runners [1]. The addition of chromium significantly enhances the material’s corrosion resistance, while nickel and molybdenum improve its toughness and strength [2,3]. The low carbon content (0.04%) provides excellent workability and crack resistance during welding and heat treatment processes [4]. In hydroelectric power generation, turbine runners face significant challenges from sediment-laden water flow [5]. Even fine particles can cause substantial damage to hydraulic turbines during operation, leading to reduced efficiency and increased maintenance costs. Additionally, since runners normally are welded to blades during manufacturing, good weldability is essential [6]. In addition to the manufacturing issues, welding repairing often drastically alters the material’s microstructure, negatively impacting its toughness [7]. Due to its superior combination of properties, 04Cr13Ni5Mo has become the preferred material for manufacturing Pelton turbine runners [8]. However, 04Cr13Ni5Mo steel exhibits strong microstructural inheritance. If coarse or mixed grains form during forging or casting, they cannot be effectively refined through subsequent heat treatment [9]. Therefore, investigating its dynamic recrystallization behavior and grain evolution is critical for manufacturing large-scale 04Cr13Ni5Mo martensitic stainless steel runner forgings with optimal microstructures.

The performance of forgings is fundamentally determined by their microstructure. Numerous researchers have investigated the microstructural evolution of martensitic stainless steels. Shirazi et al. [10] employed electron backscatter diffraction (EBSD) to examine Fe-Ni martensitic steel and found that reversed austenite grains maintained nearly identical crystallographic orientations to the original grains, demonstrating that the original grain orientations and boundaries were preserved due to the austenite memory effect. Ma et al. [11] studied commercial super martensitic stainless steel (SMSS) and discovered that excessive nitrogen content leads to the formation of Cr-rich nitrides, which significantly degrade the material’s pitting corrosion resistance. Barlow et al. [12] investigated the relationship between microstructure and grain size in two martensitic stainless steels, revealing that carbide dissolution during austenitization promotes grain growth. Salleh et al. [13] examined the effect of tempering on 440C martensitic stainless steel’s hardness and observed that secondary hardening occurring after 30 min of tempering resulted in peak hardness values.

In investigations of dynamic recrystallization mechanisms in martensitic stainless steels, numerous researchers have quantitatively examined the effects of temperature and strain parameters on dynamic recrystallization behavior. Chen et al. [14] analyzed the critical deformation conditions of a novel high-strength martensitic stainless steel (HSMSS) for aerospace bearings under 900–1150 °C at strain rates of 0.01–10 s^−1^, demonstrating that increased deformation temperature and decreased strain rate significantly promote dynamic recrystallization. Ren et al. [15] investigated the hot deformation behavior of X20Cr13 martensitic stainless steel and established a recrystallized grain size model for the same temperature and strain rate range (900–1150 °C/0.01–10 s^−1^), with model predictions showing excellent agreement with experimental results. Ebrahimi G R et al. [16] determined two critical dynamic recrystallization parameters—the critical strain and maximum dynamic softening point—for 13%Cr-containing martensitic stainless steel. Zeng et al. [17] revealed that heat-resistant martensitic stainless steel 403Nb exhibits both dynamic recovery and dynamic recrystallization under deformation conditions of 900–1150 °C and strain rates of 0.01–1 s^−1^, with dynamic recrystallization occurring preferentially at temperatures above 1000 °C and strain rates below 0.5 s^−1^. These comprehensive studies on the occurrence conditions and key parameters of dynamic recrystallization in martensitic stainless steels have established an important theoretical foundation for subsequent research in this field.

With the advancement of computer technology, finite element simulation has emerged as a powerful tool for investigating the hot working processes of martensitic stainless steels [18]. Dourandish et al. [19] employed finite element simulation to examine dynamic softening phenomena during the hot forging of martensitic stainless steels, successfully optimizing deformation processes through integration with processing maps. Vukelic et al. [20] conducted numerical simulations to study the fracture behavior of X20Cr13 martensitic steel, providing valuable theoretical insights into material fracture mechanisms.

Dynamic recrystallization behavior plays a very important role in predicting the microstructure evolution of materials. Kopp was the first to put forward the dynamic recrystallization (DRX) model of metallic materials [21]. Irani M et al. proposed the Johnson–Mehl–Avrami–Kolmogorov (JMAK) model to describe the dynamic recrystallization behavior of 100CrMnSi6 steel [22]. Zhong et al. took Incoloy 028 alloy as the object and proposed a new discontinuous dynamic recrystallization model for the initial microstructure properties [23]. Ji et al. established a dynamic recrystallization kinetics (DRX) model of 33Cr23Ni8Mn3N using the JMAK model and simulated its thermal compression based on Deform-3D. The results showed high reliability [24]. Baron et al. established the dynamic recrystallization dynamic model of ms-w1200 martensitic stainless steel, and developed the finite element simulation program, which proved the good consistency between the simulated grain size prediction and the experimental results [25]. Therefore, the establishment of dynamic recrystallization models combined with numerical simulation technology is an important means to control the microstructure evolution during metal plastic deformation.

With the ongoing trend toward manufacturing increasingly larger Pelton turbine runners under extreme service conditions, understanding and controlling the dynamic recrystallization behavior of 04Cr13Ni5Mo martensitic stainless steel has emerged as a critical research challenge in hydropower engineering. Addressing this challenge, the present study makes three significant contributions: (1) systematic investigation of hot deformation behavior under industrially relevant conditions (950–1200 °C, 0.001–0.1 s^−1^) through precision thermomechanical testing; (2) development of a novel dynamic recrystallization kinetic model specifically tailored for 04Cr13Ni5Mo steel; and (3) comprehensive validation through integrated experimental and computational approaches. The established model provides excellent predictive capability for grain size evolution during large-scale forging operations, offering a scientific foundation for manufacturing ultra-large runners with optimized microstructures. This work represents a substantial advancement over previous studies by providing quantitative process–structure relationships that enable precise microstructure control—a crucial requirement for ensuring the long-term reliability of next-generation hydropower turbines operating under extreme sediment erosion conditions.

## 2. Materials and Methods

**Figure 1:**
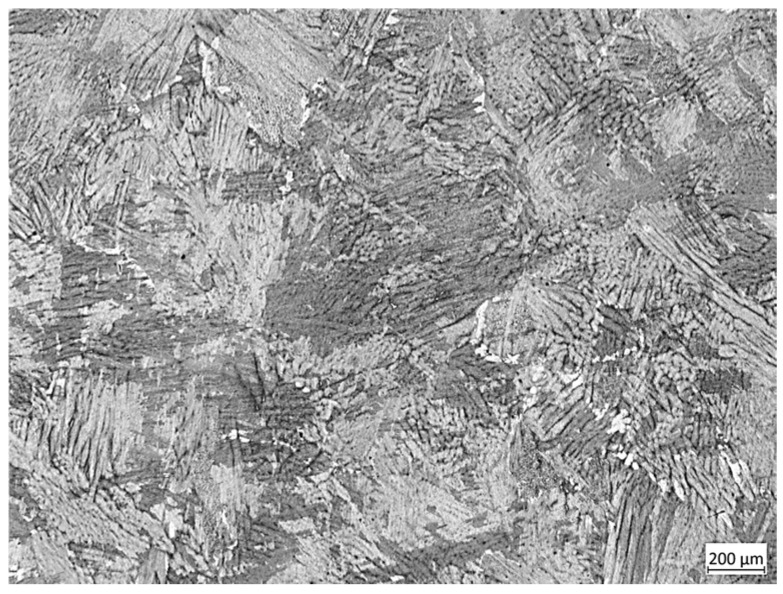
The initial microstructure of 04Cr13Ni5Mo.

**Figure 2:**
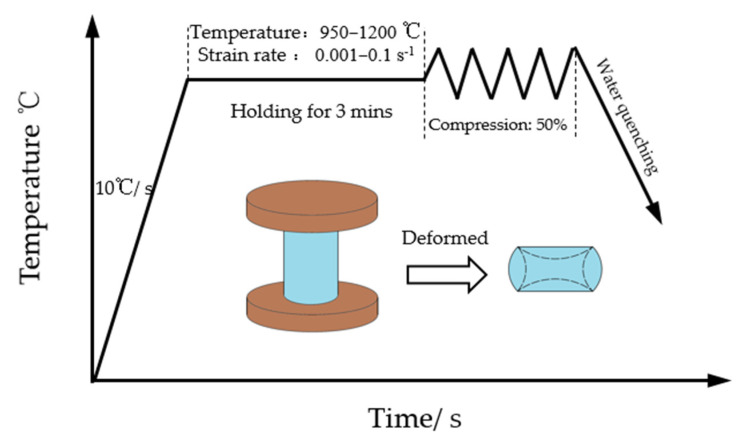
Schematic diagram of the thermal compression test.

The martensitic stainless steel 04Cr13Ni5Mo was adopted as the research object. The 04Cr13Ni5Mo test material was subjected to spectral analysis using a TW440030 Axios-XRFX fluorescence spectrometer (PANalytical, Almelo, The Netherlands), and its chemical composition (mass fraction, %) was determined as shown in Table 1. The initial grains are shown in Figure 1. The test temperatures were 950, 1000, 1050, 1100, 1150, and 1200 °C, and the strain rates were 0.001, 0.01, and 0.1 s^−1^, with a total of 18 specimens. The sample size was φ8 × 12 mm, and the pressure reduction was 50%. The compression test of 04Cr13Ni5Mo was achieved on a Gleeble-1500D Thermomechanical Simulation Tester (Dynamic Systems Inc, New York, NY, USA). The sample was heated to the specified temperature at a rate of 10 °C/s, held for 3 min, and then compressed at the set strain rate, as shown in Figure 2. After the process, it was water-cooled to retain the high-temperature structure. After compression, these specimens were cut along the axial direction by wire cutting, ground and polished with sandpaper, etched in a 4% potassium permanganate +6% ferric sulfate solution, and the microstructures of the central area of the specimens’ cross-sections were observed with a MAT200 metallographic microscope (Nikon, Langen, Germany). The average grain size of dynamic recrystallization was measured, and the dynamic recrystallization behavior was analyzed in combination with the stress–strain curve.

We established a dynamic recrystallization model of 04Cr13Ni5Mo material and wrote it into the material file of Forge^®^NxT 3.2 software. We conducted high-temperature compression tests under the same test deformation conditions. The simulation and test conditions were the same. The samples were φ8 × 12 mm cylinders. The friction conditions with the indenter were selected as water and graphite, and the heat exchange was selected as vacuum insulation.

## 3. Results

### 3.1. The Flow Stress Curve of 04Cr13Ni5Mo Stainless Steel

**Figure 3:**
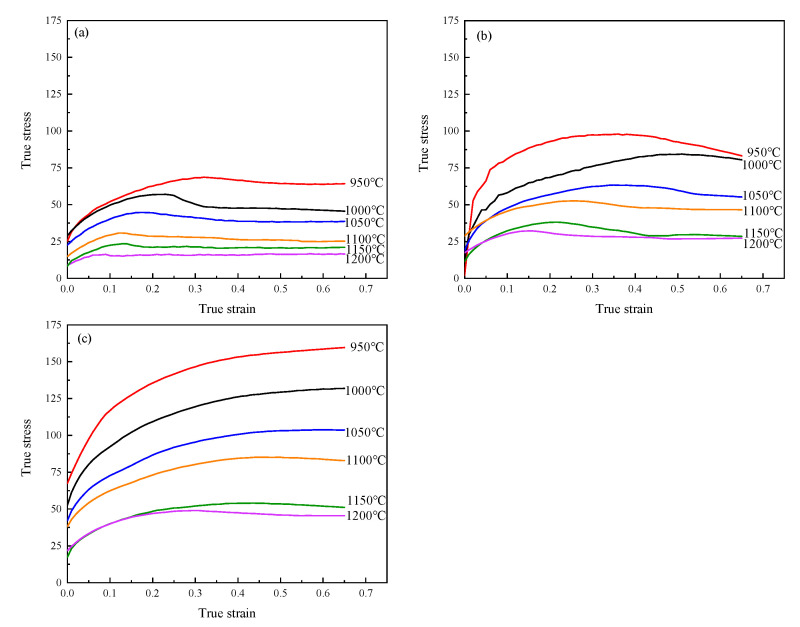
True stress-strain curve of 04Cr13Ni5Mo. (**a**) 0.001 s^−1^; (**b**) 0.01 s^−1^; (**c**) 0.1 s^−1^.

The true stress–strain curves of 04Cr13Ni5Mo stainless steel are shown in Figure 3. As can be seen from Figure 3, the flow stress curve of 04Cr13Ni5Mo stainless steel exhibits typical dynamic recrystallization (DRX) characteristics. In the initial stage of specimen compression, the stress value increases rapidly due to dislocation accumulation. When the critical stress is reached, new grains begin to form within the material through DRX, causing the stress increase rate to decelerate. During this stage, work hardening dominates, driving the stress to its peak value. In the dynamic softening stage, as new grains nucleate and grow, both DRX and dynamic recovery (DRV) become predominant. In the steady-state stage, the stress value gradually decreases with increasing strain until equilibrium is achieved between DRV, DRX, and work hardening [26].

**Figure 4:**
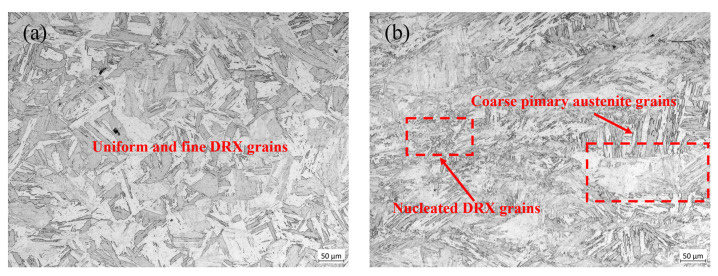
Microstructure picture of 04Cr13Ni5Mo. (**a**) 1200 °C/0.01 s^−1^; (**b**) 1000 °C/0.1 s^−1^.

As shown in Figure 1, under the condition of 1200 °C/0.01 s^−1^, the material first reached its peak stress of 32.57 MPa at a strain of 0.162. With continued deformation, DRV and DRX processes persisted within the material, leading to stress reduction until a steady-state stress of 29.87 MPa was attained. As shown in Figure 4a, it can be seen that under this condition, the grains were fine and uniform, being 91.69 μm. Under the 1000 °C/0.1 s^−1^ condition, the stress initially increased sharply with the strain, and the growth rate slowed down and gradually increased to 127.43 MPa. As shown in Figure 4b, it can be seen that both extremely coarse original grains and dynamic recrystallized grains just beginning to nucleate existed simultaneously, with dimensions of 231.66 μm and 18.52 μm, respectively.

### 3.2. Establishment of the Recrystallization Model of 04Cr13Ni5Mo Steel

#### 3.2.1. Identification Parameters of the Critical Strain Model

Dynamic recrystallization of 04Cr13Ni5Mo stainless steel is the process of forming new equiaxed crystals within the material. Dynamic recrystallization can occur only when the material deformation reaches the critical strain. Kopp et al. proposed and constructed a critical strain model for metallic materials to describe the relationship between temperature, strain rate, and critical strain [21]. Based on this, scholars such as Chen identified the parameters of the model through mathematical derivation [27]. When the material begins to recrystallize, there will be an inflection point for the work hardening rate θ [28]. The θ values were obtained through Equation (1):

(1) θ=𝜕σ/𝜕ε

Among them, σ is stress, θ is work hardening rate, and ε is strain. By differentiating the work hardening rate, the following was obtained:

(2) $$\frac{𝜕\theta }{𝜕\sigma }=\frac{𝜕\theta }{𝜕\varepsilon }\times \frac{𝜕\varepsilon }{𝜕\sigma }=\frac{𝜕\theta }{𝜕\varepsilon }\times \frac{1}{\theta }=\frac{𝜕(\ln\theta )\theta }{𝜕\varepsilon }$$

The value of critical strain was obtained by drawing the 𝜕(lnθ)θ/𝜕ε−ε curve, and the relationship between lnθ and ε was expressed by Equation (3):

(3) $$\ln\theta =A+B\varepsilon +C\varepsilon^{2}+D\varepsilon^{3}$$

Taking the derivative of the above equation yields Equation (4):

(4) $$𝜕(\ln\theta )/𝜕\varepsilon =B+2C\varepsilon +3D\varepsilon^{2}$$

Therefore, the critical strain $\varepsilon_{c}$ was obtained through Equation (5):

(5) $$\varepsilon_{c}=-C/3D$$

**Figure 5:**
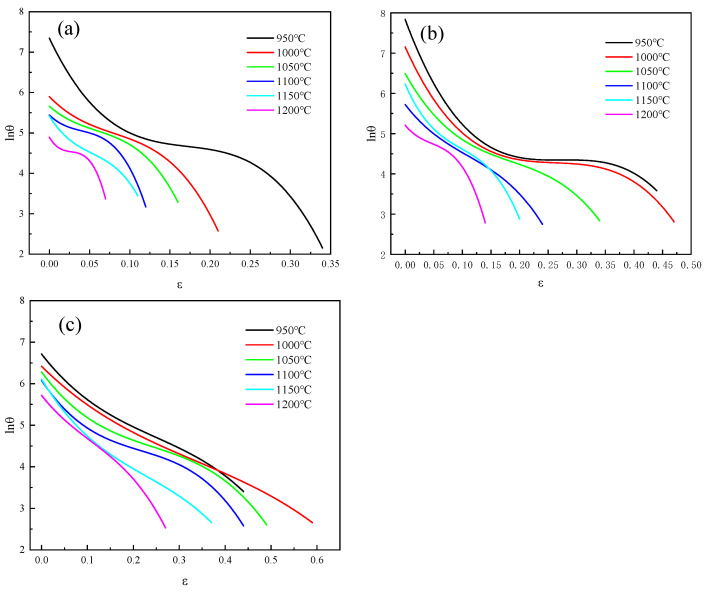
lnθ−ε fitting curve graph. (**a**) 0.001 s^−1^; (**b**) 0.01 s^−1^; (**c**) 0.1 s^−1^.

Figure 5 shows the cubic polynomial fitting curves under different deformation conditions. Among them, the lnθ−ε curve under the condition of 950 °C/0.01 s^−1^ is shown in Figure 5b. It could be seen that when the strain increased, lnθ gradually decreased and approached a certain value, and then dropped rapidly. Its fitting curve was:

(6) $$\ln\theta =7.912-43.11\varepsilon +200.003\varepsilon^{2}-375.577\varepsilon^{3}$$

**Figure 6:**
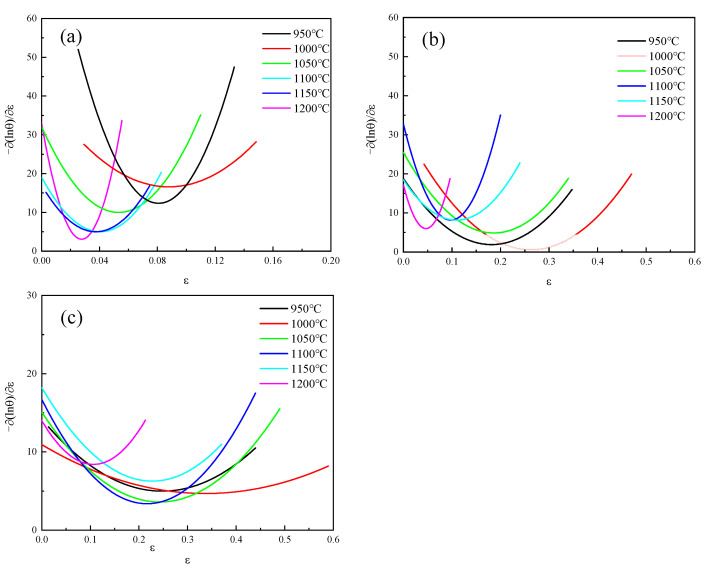
−𝜕(ln(θ))/𝜕ε−ε fitting curve graph. (**a**) 0.001 s^−1^; (**b**) 0.01 s^−1^; (**c**) 0.1 s^−1^.

Figure 6 shows the curve graph of  −𝜕(ln(θ))/𝜕ε−ε fitting under different deformation conditions. As shown in Table 2, the lnθ curve fitting parameters and critical strain of 04Cr13Ni5Mo stainless steel under different deformation conditions were presented.

The peak strain model formula of 04Cr13Ni5Mo stainless steel was as follows:

(7) $$\varepsilon_{c}=a\cdot \varepsilon_{p}$$

(8) $$\varepsilon_{p}=A_{P}\cdot {\dot{\varepsilon }}^{m_{p}}\cdot \exp(\frac{Q_{P}}{RT})$$

In the formula, $\varepsilon_{p}$ is the peak strain, $\varepsilon_{c}$ is the critical strain, $\dot{\varepsilon }$ is the strain rate, $Q_{P}$ is the activation energy, T is the temperature, and the rest are constants.

**Figure 7:**
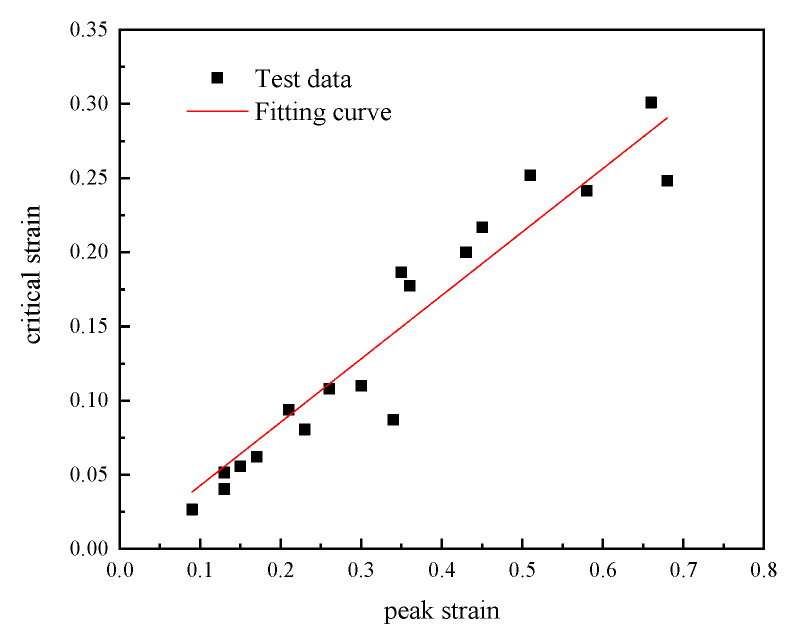
Fitting curves of $\;\varepsilon_{p}-\varepsilon_{c}$ at different temperatures and strain rates.

The peak strain of 04Cr13Ni5Mo under different deformation conditions were shown in Table 3. As shown in Figure 7, the peak strain and critical strain of 04Cr13Ni5Mo stainless steel under various deformation conditions were linearly fitted to obtain a slope of a=0.4274, and the correlation coefficient was $R^{2}=0.983$.

Taking the logarithm of Equation (8) yields:

(9) $$\ln\varepsilon_{p}=\ln A_{P}+m_{p}\ln\dot{\varepsilon }+\frac{Q_{P}}{RT}$$

**Figure 8:**
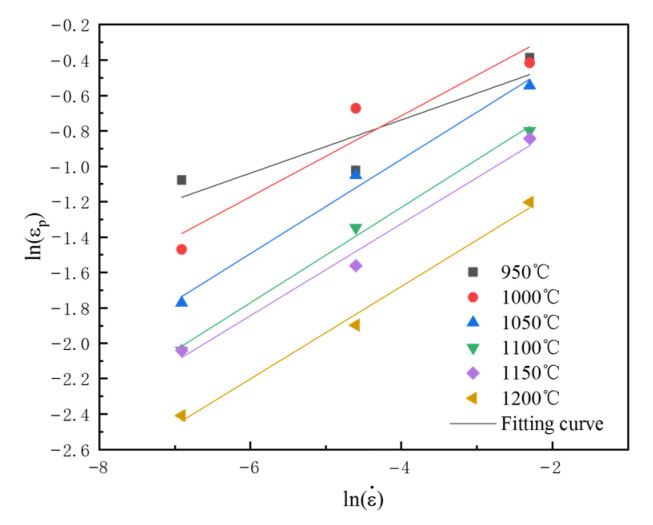
Fitting curves at different temperatures.

When the temperature is constant, $\ln A_{P}+Q_{p}/RT$ is constant. A linear analysis of $\ln(\varepsilon_{p})-\ln(\dot{\varepsilon })$ was made, as shown in Figure 8, and its slope is $m_{p}$. The average value of $m_{p}$ at different temperatures was calculated, which was 0.232.

When the strain rate was constant, $\ln A_{P}+m_{p}\ln\dot{\varepsilon }$ was constant. Therefore, the slope of $\ln\varepsilon_{p}-1/T$ was $Q_{P}/R$ through linear analysis. The average value of $Q_{P}/R$ at different temperatures was calculated, which was 7353.562 J/mol, and $Q_{P}=61137.514$ J/mol.

We substituted $m_{p}$ and $Q_{P}$ into Equation (8), calculated the value of $A_{P}$ under each deformation condition, and found its arithmetic average value was 0.00352.

The critical strain calculation formula could be expressed as follows:

(10) $$\varepsilon_{c}=0.4274\varepsilon_{p}$$

(11) $$\varepsilon_{p}=0.00352{\dot{\varepsilon }}^{0.232}\cdot \exp(\frac{61137.514}{RT})$$

#### 3.2.2. Identification of Parameters of the Dynamic Recrystallization Model of 04Cr13Ni5Mo Martensitic Stainless Steel

Common methods for establishing the volume fraction of dynamic recrystallization of materials include the metallographic method, the energy method, and the true stress–strain curve method [29]. However, the metallographic method has relatively high requirements for the metallographic cross-section and corrosion results, and the energy method is inconvenient when measuring deformation energy storage. Therefore, the true stress–strain curve method was selected to determine the dynamic recrystallization volume fraction of 04Cr13Ni5Mo stainless steel.

The relationship between the percentage of dynamic recrystallization and stress was [30]:

(12) $$X_{DRX}=\frac{\sigma_{drv}-\sigma }{\sigma_{sat}-\sigma_{ss}}$$

**Figure 9:**
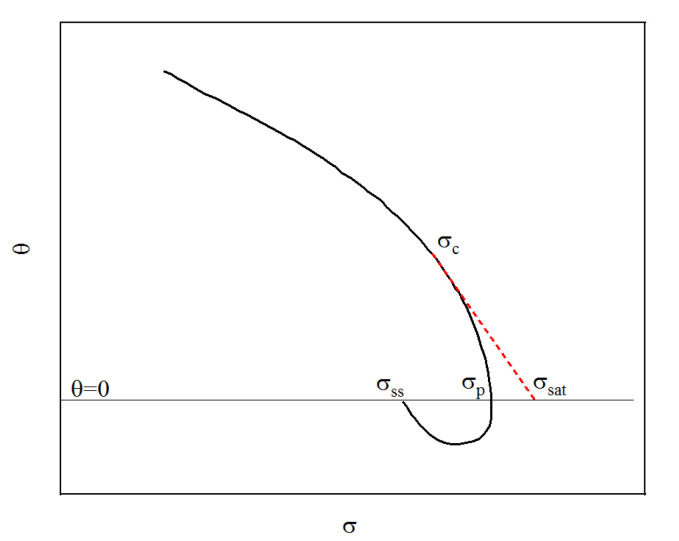
Typical θ−σ curve.

In the formula, $\sigma_{drv}$ represents the flow stress value considering only dynamic recovery (DRV), that is, the stress value obtained from the dynamic recovery curve of the material; $\sigma_{sat}$ represents the saturation stress of the DRV curve, and $\sigma_{ss}$ represents steady-state stress. As shown in Figure 9, The $\sigma_{sat}$ could be obtained through the θ−σ curve, and its inflection point was the critical stress point. A tangent line was drawn from this inflection point and intersects with θ=0, and the intersection point was the sought $\sigma_{sat}$; $\sigma_{drv}$ could be obtained by Equation (13):

(13) $$\sigma_{drv}=\sigma_{sat}+(\sigma_{c}-\sigma_{sat})\exp(\frac{(\varepsilon -\varepsilon_{c})\theta_{c}}{\sigma_{c}-\sigma_{sat}})$$

**Figure 10:**
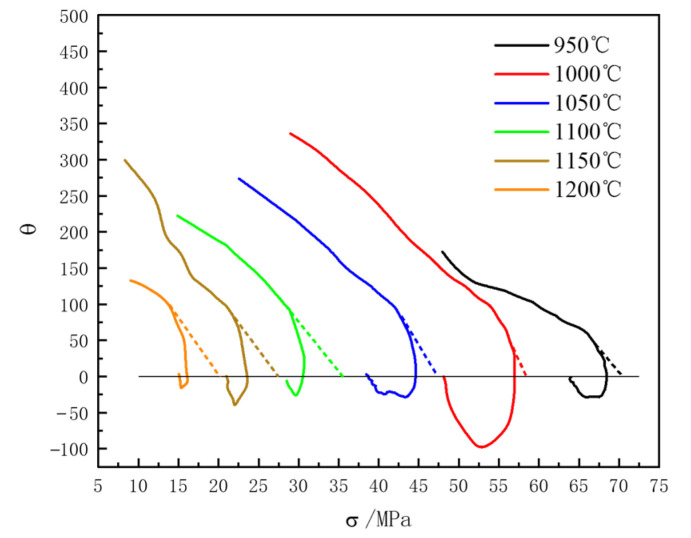
Curve θ−σ graphs of 04Cr13Ni5Mo stainless steel at different temperatures at a strain rate of 0.001 s^−1^.

The θ−σ curve of 04Cr13Ni5Mo stainless steel under the 0.001 s^−1^ condition is shown in Figure 10. The θ−σ curves under the deformation conditions of 0.01 and 0.1 s^−1^ were processed by the same method, and the obtained $\sigma_{sat}$ data are shown in Table 4.

**Figure 11:**
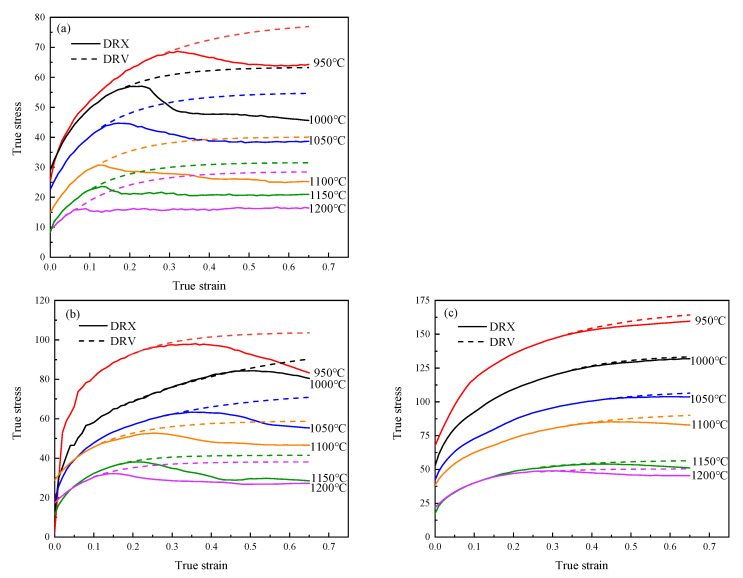
The saturation stress curves of 04Cr13Ni5Mo stainless steel at different temperatures and strain rates. (**a**) 0.001 s^−1^; (**b**) 0.01 s^−1^; (**c**) 0.1 s^−1^.

The saturation stress curves of 04Cr13Ni5Mo stainless steel under different conditions were plotted according to Equation (13), as shown in Figure 11.

**Figure 12:**
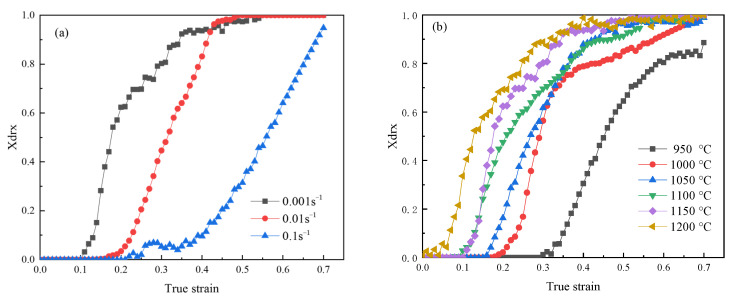
The dynamic recrystallization percentage of 04Cr13Ni5Mo stainless steel under different deformation conditions. (**a**) 950 °C; (**b**) 0.001 s^−1^.

The percentage of dynamic recrystallization was calculated based on Equation (12). Through Figure 12, it was the percentage of dynamic recrystallization under different deformation conditions.

The characteristic strain $\varepsilon_{0.5}$ of dynamic recrystallization is the strain at which 50% of the volume within the material undergoes dynamic recrystallization. Its value is shown in Table 5.

The dynamic recrystallization kinetic equation proposed by Kopp is expressed by the following formula:

(14) $$\left\{\begin{array}{l} X_{DRX}=1-\exp\left[-k_{d}{\left(\frac{\varepsilon -\varepsilon_{c}}{\varepsilon_{0.5}-\varepsilon_{c}}\right)}^{n_{DRX}}\right]\\ \varepsilon_{0.5}=A_{0.5}{\dot{\varepsilon }}^{m_{0.5}}\cdot \exp(\frac{Q_{0.5}}{RT}) \end{array}\right.$$

In the formula, $X_{DRX}$ is the volume fraction of dynamic recrystallization, $\varepsilon_{0.5}$ represents the characteristic strain of 14Cr1Mo steel under different conditions, $\varepsilon_{c}$ represents the strain of critical 14Cr1Mo steel under different conditions, and $k_{d}$, $n_{DRX}$, $A_{0.5}$, $m_{0.5}$, and $Q_{0.5}$ are constants.

The characteristic strain expression $\varepsilon_{0.5}$ is similar to the peak strain Formula (8). By using the same method, $m_{0.5}=0.237$, $Q_{0.5}=24,325.793$ J/mol, and $A_{0.5}=0.1354$ were calculated. The characteristic strain model of 04Cr13Ni5Mo was obtained:

(15) $$\varepsilon_{0.5}=0.0478{\dot{\varepsilon }}^{0.0612}\cdot \exp(\frac{24466.766}{RT})$$

When $\varepsilon =\varepsilon_{0.5}$, $X_{DRX}$ = 0.5, and $k_{d}$ = ln2 = 0.693. We substituted $k_{d}$ = ln2 = 0.693 into Equation (14), and took the natural logarithm of the formula to obtain:

(16) $$\left\{\begin{array}{l} \ln(-\ln(1-X_{DRX}))=\ln0.693+n_{DRX}\cdot \ln(\frac{\varepsilon -\varepsilon_{c}}{\varepsilon_{0.5}-\varepsilon_{c}})\\ \ln\varepsilon_{0.5}=\ln A_{0.5}+m_{0.5}\ln\dot{\varepsilon }+\cdot \frac{Q_{0.5}}{RT} \end{array}\right.$$

Therefore, it could be obtained:

(17) $$n_{DRX}=\frac{𝜕\ln[-\ln(1-X_{DRX})]}{𝜕\ln(\frac{\varepsilon -\varepsilon_{c}}{\varepsilon_{0.5}-\varepsilon_{c}})}$$

**Figure 13:**
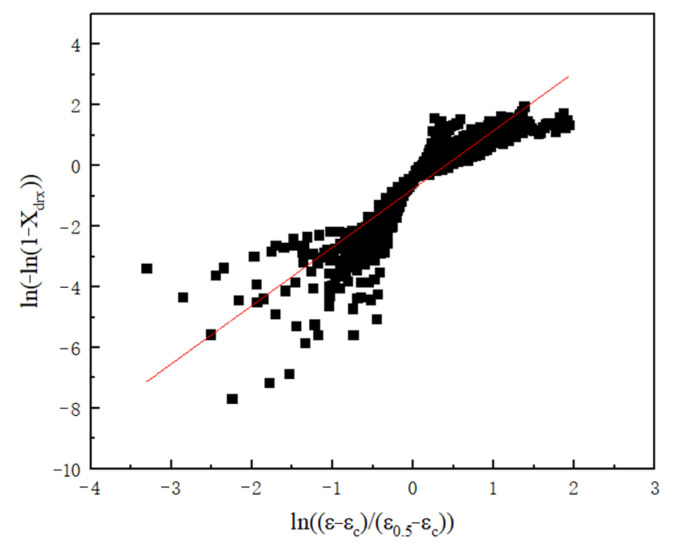
The fitting curves of $\ln[-\ln(1-X_{DRX})]$ and $\ln[(\varepsilon -\varepsilon_{c})/(\varepsilon_{0.5}-\varepsilon_{c})]$.

A linear fitting was performed on $\ln[-\ln(1-X_{DRX})]$ and $\ln[(\varepsilon -\varepsilon_{c})/(\varepsilon_{0.5}-\varepsilon_{c})]$, and its slope was the value of $n_{DRX}$, $n_{DRX}=1.923$, through Figure 13.

The dynamic recrystallization percentage model of 04Cr13Ni5Mo steel is:

(18) $$\left\{\begin{array}{l} X_{DRX}=1-\exp\left[-0.693{\left(\frac{\varepsilon -\varepsilon_{c}}{\varepsilon_{0.5}-\varepsilon_{c}}\right)}^{1.923}\right]\\ \varepsilon_{0.5}=0.1354{\dot{\varepsilon }}^{0.237}\cdot \exp(\frac{24,325.793}{RT}) \end{array}\right.$$

The 04Cr13Ni5Mo hot compression specimen was cut along the axial direction, embedded with the specimen, corroded with ferric chloride hydrochloric acid aqueous solution, and the grain size was measured. The grain sizes under different deformation conditions are shown in the Table 6.

The grain size of dynamic recrystallization of 04Cr13Ni5Mo steel is:

(19) $$D_{DRX}=A_{D}{\dot{\varepsilon }}^{m_{D}}\cdot \exp(\frac{Q_{D}}{RT})$$

The expression of this formula was in the same form as the peak strain model, and it was easy to obtain $A_{D}=6433.19$, $m_{D}=-0.258$, and $Q_{D}=-68,222.3$ J/mol. Therefore, the expression for the grain size of the dynamic recrystallization of 04Cr13Ni5Mo is:

(20) $$D_{DRX}=6433.19{\dot{\varepsilon }}^{-0.258}\cdot \exp(\frac{-68,222.3}{RT})$$

#### 3.2.3. Verification of the Dynamic Recrystallization Kinetic Model of 04Cr13Ni5Mo Steel Based on Finite Element Simulation

The finite element method has been applied to the analysis of microstructure evolution during the metal forming process [31]. The hot compression tests of 04Cr13Ni5Mo under different temperatures and strain rates were simulated under the same conditions, and the grain size data at the center of the axial cross-section were obtained, as shown in Table 7.

**Figure 14:**
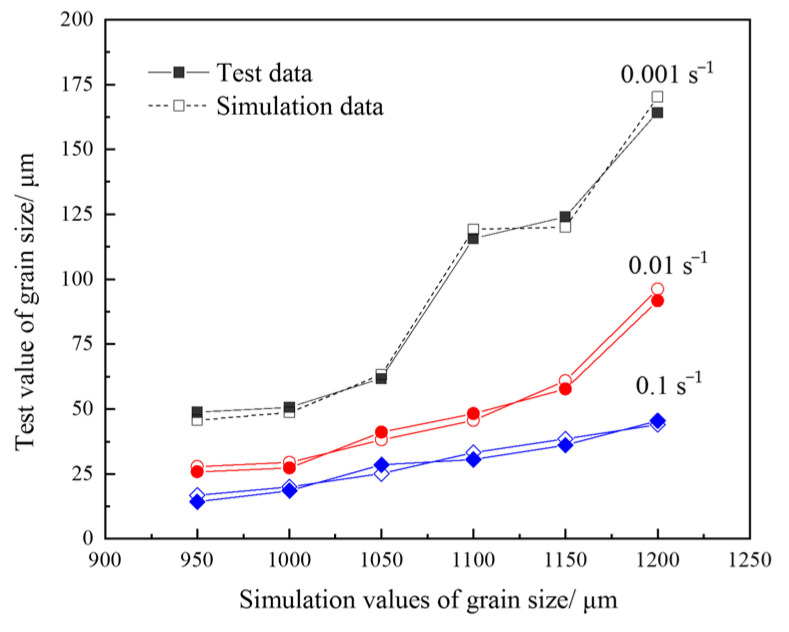
Comparison of grain sizes between finite element simulation and test value.

Figure 14 shows the comparison between the grain size of 04Cr13Ni5Mo in the finite element simulation and the experimental data. It can be seen that the simulation results are highly consistent with the actual grain size. Under different conditions, the correlation coefficient between its simulation and the experimental grain size was relatively high, which was 0.997. It was easy to see that when the deformation temperature was higher than 1100 °C, the growth rate of its grain size was relatively large.

**Figure 15:**
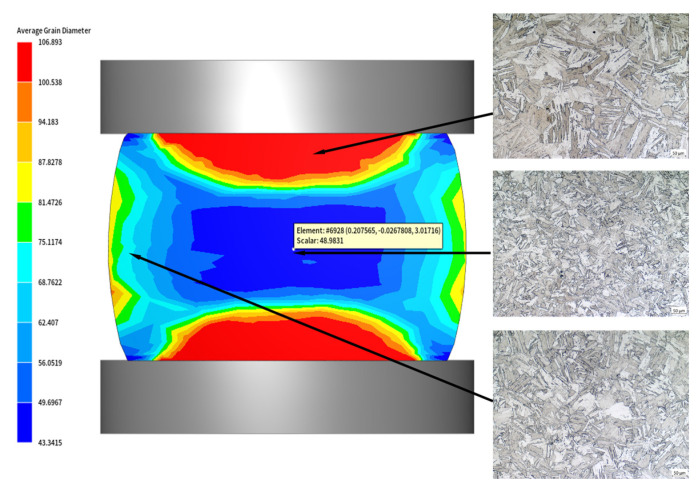
Comparison of simulated and measured grain sizes in different regions of compressed specimens under 1100 °C/0.01 s^−1^ conditions.

Figure 15 shows the comparison between the simulation cloud image at 1100 °C/0.01 s^−1^ and the metallographic structure. The grains size in the upper and lower difficult to deform zones were the largest, which were 106.89 μm. The grains in the small circumferential deformation zone were very small, being 68.76 μm. The grains in the central large deformation zone were the finest, at 48.98 μm. By observing and measuring the actual microscopic grain structure of the sample, it could be found that the grain sizes of the three deformation zones were 103.74 μm, 66.43 μm, and 48.18 μm, respectively, which were highly consistent with the simulation results. Therefore, the model could be used to predict the dynamic recrystallization structure evolution of 04Cr13Ni5Mo steel, providing theoretical guidance for optimizing the forming process of runner forgings and refining the grains.

### 3.3. The Evolution Law of Dynamic Recrystallization Structure of 04Cr13Ni5Mo Stainless Steel

**Figure 16:**
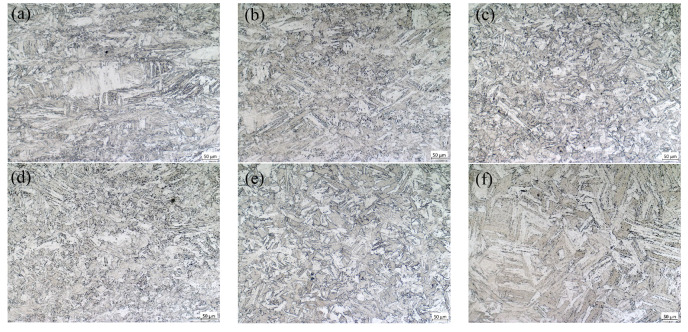
The microstructure of 04Cr13Ni5Mo under different conditions. (**a**) 950 °C/0.1 s^−1^; (**b**) 1050 °C/0.1 s^−1^; (**c**) 1150 °C/0.1 s^−1^; (**d**) 1100 °C/0.1 s^−1^; (**e**) 1100 °C/0.01 s^−1^; (**f**) 1100 °C/0.001 s^−1^.

Dynamic recrystallization is the formation of new strain-free grains through nucleation and growth processes under the influence of temperature and strain rate, resulting in the refinement of grain size [32]. It could be seen from Figure 16 that under the condition of 950 °C/0.1 s^−1^, a large number of grains were significantly elongated, and only fine grains undergoing dynamic recrystallization occurred locally, with a size of 14.23 μm. When the temperature rose to 1050 °C, the proportion of dynamic recrystallization structure increased, and the grain size of its dynamic recrystallization was 18.52 μm. The temperature further rose to 1150 °C, dynamic recrystallization occurred completely, and the grain size was 36.15 μm. This suggests that the rise in temperature will provide adequate activation energy for recrystallization, facilitate nucleation of recrystallization and migration of grain boundaries, enhance the completeness of recrystallization, and simultaneously accelerate grain growth [33].

At 1100 °C/0.1 s^−1^, its dynamic recrystallization had completely occurred, and the grain size was 30.54 μm. When the strain rate decreased to 0.01 s^−1^, the grain size increased to 48.18 μm. When the strain rate further decreased to 0.001 s^−1^, the grain size increased to 115.54 μm. This is because when the temperature has provided a sufficiently high activation energy for dynamic recrystallization, prolonging the plastic deformation time will cause the dynamic recrystallized grains that have occurred to regrow into coarse grains.

## 4. Conclusions

As a key component of hydraulic turbines, the microstructural and mechanical properties of runner forgings are necessary to ensure safe operation under harsh conditions. To effectively predict and control the microstructure and grain size within the rotor, this paper studies the dynamic recrystallization behavior and microstructure evolution of 04Cr13Ni5Mo steel during the hot forming process, based on the dynamic recrystallization model of 04Cr13Ni5Mo steel and finite element software. This study provides a reliable theoretical basis for optimizing the thermal processing parameters of the runner and manufacturing large-scale runners with an excellent microstructure and properties. The main conclusions are as follows:

The 04Cr13Ni5Mo steel exhibits typical dynamic recrystallization characteristics at 950–1200 °C and 0.001–0.1 s^−1^. The stress increases to the peak stress with increasing strain, and then gradually decreases and stabilizes. At 950 °C, the dynamic recrystallization of this material was insufficient, and the microstructure consisted of both coarse original grains and newly formed recrystallized grains. When the temperature rose to 1150 °C, dynamic recrystallization occurred fully, and the microstructure was relatively uniform.

Based on the experimental data and microstructure observations of 04Cr13Ni5Mo steel, the dynamic recrystallization kinetic model was established:

$$\left\{\begin{array}{l} X_{DRX}=1-\exp\left[-0.693{\left(\frac{\varepsilon -\varepsilon_{c}}{\varepsilon_{0.5}-\varepsilon_{c}}\right)}^{1.923}\right]\\ \varepsilon_{0.5}=0.1354{\dot{\varepsilon }}^{0.237}\cdot \exp(\frac{24,325.793}{RT})\\ D_{DRX}=6433.19{\dot{\varepsilon }}^{-0.258}\cdot \exp(\frac{-68,222.3}{RT}) \end{array}\right.$$

In order to verify the accuracy of the model, the finite element software Forge was redeveloped, and the established dynamic recrystallization model was embedded in the software. All of the hot compression tests were simulated under the same conditions. Compared with the grain size in the central area of the axial section measured by the simulation and the test, the correlation coefficient r was 0.997. The compression test of 04Cr13Ni5Mo steel at 100 C/0.01 s was simulated. The simulated grain sizes of different deformation zones are compared with the grain sizes measured by corresponding metallographic images. The simulated grain size is 48.98 μm, and the actual grain size is 48.18 μm. The simulation results are in good agreement with the experimental results, indicating that the established model has high accuracy. This work provides theoretical guidance for optimizing the forming process and refining the grain of runner forgings.

## Figures and Tables

**Table 1:** Chemical composition of 04Cr13Ni5Mo (mass fraction, %).

Chemical Composition	C	Si	Mn	S	P	Cr	Ni	Mo	Cu
04Cr13Ni5Mo	0.033	0.36	0.71	0.003	0.018	12.9	4.9	0.6	0.053

**Table 2:** Critical strain of 04Cr13Ni5Mo under different deformation conditions.

Temperature/°C	Strain Rate/s^−1^	Fitting Parameters					Critical Strain
		A	B	C	D	R^2^	
950	0.001	7.346	−42.068	230.896	−886.542	0.973	0.087
	0.01	7.912	−43.11	200.003	−375.577	0.991	0.178
	0.1	6.712	−14.197	37.15	−49.936	0.99	0.248
1000	0.001	5.897	−20.517	171.917	−712.326	0.993	0.080
	0.01	7.255	−35.074	139.821	−185.035	0.978	0.252
	0.1	6.506	−13.272	30.336	−33.603	0.952	0.301
1050	0.001	5.703	−22.489	280.982	−1509.735	0.988	0.062
	0.01	6.491	−25.485	110.554	−197.727	0.973	0.186
	0.1	6.120	−10.538	23.013	−31.795	0.956	0.241
1100	0.001	5.440	−19.078	350.37	−2909.149	0.986	0.040
	0.01	5.764	−21.638	134.05	−413.944	0.953	0.108
	0.1	6.071	−16.707	61.514	−94.637	0.986	0.217
1150	0.001	5.762	−40.591	653.031	−4234.564	0.988	0.051
	0.01	6.334	−41.949	391.429	−1392.689	0.969	0.094
	0.1	6.179	−21.348	78.129	−130.293	0.936	0.200
1200	0.001	5.002	−45.729	1919.919	−24,064.599	0.973	0.027
	0.01	5.477	−40.078	740.987	−4440.765	0.944	0.056
	0.1	5.766	−17.008	86.428	−261.066	0.988	0.110

**Table 3:** Peak strain of 04Cr13Ni5Mo stainless steel under different deformation conditions.

Peak Strain	950 °C	1000 °C	1050 °C	1100 °C	1150 °C	1200 °C
0.001 s^−1^	0.34	0.23	0.17	0.13	0.13	0.09
0.01 s^−1^	0.36	0.51	0.35	0.26	0.21	0.15
0.1 s^−1^	0.68	0.66	0.58	0.45	0.43	0.30

**Table 4:** Steady-state stresses of 04Cr13Ni5Mo stainless steel under different deformation conditions.

Steady-State/MPa	950 °C	1000 °C	1050 °C	1100 °C	1150 °C	1200 °C
0.001 s^−1^	70.887	58.397	47.907	35.459	27.573	19.995
0.01 s^−1^	103.951	100.647	74.146	58.939	41.447	38.173
0.1 s^−1^	169.665	135.038	107.931	92.244	56.877	52.48

**Table 5:** Characteristic strains of 04Cr13Ni5Mo steel under different deformation conditions.

Characteristic Strains	950 °C	1000 °C	1050 °C	1100 °C	1150 °C	1200 °C
0.001 s^−1^	0.45	0.29	0.27	0.21	0.17	0.12
0.01 s^−1^	0.58	0.62	0.54	0.34	0.31	0.21
0.1 s^−1^	0.61	0.58	0.59	0.56	0.53	0.40

**Table 6:** Grain size of 04Cr13Ni5Mo stainless steel under different deformation conditions (unit, μm).

Grain Size	0.001 s^−1^	0.01 s^−1^	0.1 s^−1^
950 °C	48.68	25.86	14.23
1000 °C	50.62	27.27	18.52
1050 °C	61.63	41.05	28.51
1100 °C	115.54	48.18	30.54
1150 °C	123.89	57.74	36.15
1200 °C	164.11	91.69	45.49

**Table 7:** Grain size simulation calculation of 04Cr13Ni5Mo stainless steel under different conditions (unit, μm).

Grain Size	0.001 s^−1^	0.01 s^−1^	0.1 s^−1^
950 °C	45.73	27.89	16.78
1000 °C	48.64	29.43	20.04
1050 °C	63.27	38.17	25.14
1100 °C	119.14	45.62	33.27
1150 °C	120.04	60.86	38.47
1200 °C	170.25	96.18	43.98

## Data Availability

The original contributions presented in this study are included in the article. Further inquiries can be directed to the corresponding author.
